# Regulation of osteoblast development by Bcl-2-associated athanogene-1 (BAG-1)

**DOI:** 10.1038/srep33504

**Published:** 2016-09-16

**Authors:** Joanna Greenhough, Emmanouil S. Papadakis, Ramsey I. Cutress, Paul A. Townsend, Richard O. C. Oreffo, Rahul S. Tare

**Affiliations:** 1 Centre for Human Development, Stem Cells and Regeneration, Institute of Developmental Sciences, University of Southampton, Southampton SO16 6YD, United Kingdom; 2 Cancer Research UK Centre Cancer Sciences Unit, Somers Building, University of Southampton, Southampton SO16 6YD, United Kingdom; 3 Institute of Cancer Sciences, Manchester Cancer Research Centre, University of Manchester, Manchester M13 9WL, United Kingdom

## Abstract

BCL-2-associated athanogene-1 (BAG-1) is expressed by osteoblast-lineage cells; early embryonic lethality in *Bag-1* null mice, however, has limited the investigation of BAG-1 function in osteoblast development. In the present study, bone morphogenetic protein-2/BMP-2-directed osteogenic differentiation of bone marrow stromal cells (BMSCs) of *Bag-1*^+/−^ (heterozygous) female mice was decreased significantly. Genes crucial for osteogenic differentiation, bone matrix formation and mineralisation were expressed at significantly lower levels in cultures of *Bag-1*^+/−^ BMSCs supplemented with BMP-2, while genes with roles in inhibition of BMP-2-directed osteoblastogenesis were significantly upregulated. 17-β-estradiol (E2) enhanced responsiveness of BMSCs of wild-type and *Bag-1*^+/−^ mice to BMP-2, and promoted robust BMP-2-stimulated osteogenic differentiation of BMSCs. BAG-1 can modulate cellular responses to E2 by regulating the establishment of functional estrogen receptors (ERs), crucially, via its interaction with heat shock proteins (HSC70/HSP70). Inhibition of BAG-1 binding to HSC70 by the small-molecule chemical inhibitor, Thioflavin-S, and a short peptide derived from the C-terminal BAG domain, which mediates binding with the ATPase domain of HSC70, resulted in significant downregulation of E2/ER-facilitated BMP-2-directed osteogenic differentiation of BMSCs. These studies demonstrate for the first time the significance of BAG-1-mediated protein-protein interactions, specifically, BAG-1-regulated activation of ER by HSC70, in modulation of E2-facilitated BMP-2-directed osteoblast development.

Endochondral ossification, the process responsible for formation of majority of the bones that make up the skeleton, involves deposition of bone matrix by osteoblasts on intermediary cartilaginous primordia, which serve as scaffolds on which bones are subsequently built. Postnatally, endochondral ossification contributes to the longitudinal growth of bones and healing of bone fractures. Disruption of endochondral bone development results in a heterogeneous group of genetic diseases known as skeletal dysplasias that are characterised predominantly by short-limb dwarfism and, in certain chondrodysplasias, prenatal death^1^. Furthermore, dysregulation of early skeletal development is directly linked to diseases of the adult skeleton such as osteoporosis^2^,^3^. Thus, improved appreciation of skeletal development will enhance our understanding of musculoskeletal diseases and inform the development of novel strategies for replacement/regeneration of skeletal tissues.

The role of molecular chaperones and their co-chaperones in health and disease has attracted significant attention in recent years. Bcl-2-associated athanogene-1 (BAG-1), the founding member of the BAG-family of co-chaperones, is a multifunctional protein, capable of regulating cell proliferation, motility, differentiation and apoptosis^4^,^5^. Murine cells express two BAG-1 isoforms, BAG-1L (50 kDa) and BAG-1S (32 kDa), which are generated using alternate translation initiation sites in a single *Bag-1* mRNA transcript^6^. During murine development, expression of *Bag-1* mRNA is detected in several organs, with highest expression observed in cartilaginous tissues of the developing mouse embryo^7^. In long bones of postnatal mice, expression of BAG-1 is detected in articular and growth plate chondrocytes, in addition to osteoblast-lineage cells, namely bone marrow stromal cells (BMSCs), osteoblasts and osteocytes^8^,^9^. Remarkably, expression of BAG-1 is upregulated in human osteoarthritic cartilage compared to normal articular cartilage, most likely in response to the disruption of chondrocyte homeostasis in osteoarthritis^10^.

BAG-1 has been demonstrated to play important roles in the protection of mammalian chondrocytes against apoptosis induced by endoplasmic reticulum stress and heat shock, and in the regulation of expression of chondrogenic markers^9^,^11^. In contrast, to date, no studies have investigated the role of *Bag-1* in osteoblast development. Gene knockout mouse models have proved pivotal in the analyses of bone development. Significant apoptosis in the embryonic liver and brain, along with defective haematopoiesis and neuronal cell differentiation have been identified as the major causes of death in *Bag-1* null mice between E (embryonic day) 12.5 and E13.5 of gestation^12^. Early embryonic lethality in *Bag-1* null mice has limited the investigation of the role of BAG-1 in bone development. This is primarily because vascular invasion of the calcified hypertrophic cartilage, resulting in the recruitment of osteoclasts and osteoblasts for the gradual replacement of the cartilaginous matrix with bone, occurs between E14.5 and E15.5^13^. Mice heterozygous for the *Bag-1* gene (i.e. containing one functional *Bag-1* allele) are not embryonic lethal and survive into adulthood^14^, thereby allowing investigation of the effect of *Bag-1* haploinsufficiency on osteoblast differentiation and bone development.

BAG-1 interacts with a diverse array of molecular targets, namely the 70-kDa heat shock chaperone proteins (HSC70/HSP70), RAF-1 kinase, components of the ubiquitylation/proteasome machinery and nuclear hormone receptors (NHRs), to regulate gene transcription and molecular signalling crucial for cell proliferation, differentiation and apoptosis^15^. Binding of BAG-1 to HSC70/HSP70 has now been acknowledged to be vital for most functions of BAG-1, including the effects of BAG-1 on NHRs^16^,^17^. The carboxy terminus BAG domain is composed of three alpha helices, which facilitate binding between BAG-1 and the amino terminal ATPase domain of HSC70/HSP70^18^. Helices 2 and 3 are involved in electrostatic interactions with the ATPase domain of HSC70/HSP70; helix 1 is not directly involved in the binding process and contributes to the intramolecular interactions that stabilise the overall structure of the BAG domain^19^. A very small region comprising of 8 amino acid residues in helix 2 of the BAG domain has been shown to be vital for binding of BAG-1 to HSC70^20^.

NHRs have been recognised as key regulators of cellular function and BAG-1 has been shown to regulate the functions of diverse NHRs, namely the glucocorticoid receptor, androgen receptor, estrogen receptors (ERs), retinoic acid receptor and vitamin D3 receptor^21^. NHRs (in their non-native states) interact with the central substrate/peptide-binding domain of the heat shock chaperone proteins and undergo a series of steps in the (re)folding/activation process to achieve correct conformations that facilitate binding of the NHRs to respective hormones^22^,^23^. The ATPase domain of HSC70/HSP70 regulates substrate binding through cycles of ATP binding and hydrolysis; substrates interact transiently with the ATP-bound form of HSC70/HSP70, while hydrolysis of ATP enables the substrates to bind the ADP-bound form of HSC70/HSP70 with high affinity^24^. Release of ADP and subsequent binding of ATP, referred to as nucleotide exchange, permits the release of the refolded substrates^24^.

BAG-1 interacts with the ATPase domain of HSC70/HSP70 and functions as a nucleotide exchange factor in the activation cycle^25^. Hence, by stimulating nucleotide exchange, BAG-1 regulates the dynamics of complex assembly crucial for the establishment and release of the functional NHRs prior to hormone binding. Thus, BAG-1, through its interaction with HSC70/HSP70, regulates the activation of NHRs, including ERs, and can play an important role in the modulation of cellular responses to steroid hormones, such as estrogen/17-β-estradiol (E2). Moreover, after hormone binding, BAG-1 is able to influence receptor-mediated transcription of the nuclear hormone-responsive genes e.g. BAG-1 has been shown to interact with and stimulate the activity of both ERα and ERβ, and enhance E2-dependent transcription in breast cancer cells^26^.

Estrogen exerts a protective effect on bone and has been demonstrated to enhance the osteogenic differentiation of osteoprogenitor cells stimulated by bone morphogenetic proteins-2/4 (BMP-2/4)^27^,^28^. However, the significance of BAG-1-regulated activation of ER by HSC70 in the modulation of E2-facilitated BMP-directed osteoblast differentiation has not been elucidated to date. Since BAG-1 interacts with most of the molecular targets in cells via HSC70/HSP70, disruption of binding of BAG-1 to the heat shock proteins leads to loss of BAG-1-mediated protein-protein interactions. Screening of compound collections (obtained from the National Cancer Institute Developmental Therapeutics Programme, Rockville, MD) for identification of small molecule chemical inhibitors of the interaction between BAG-1 and heat shock proteins resulted in the identification of NSC71948, which was found to be structurally similar to Thioflavin-S^29^. Furthermore, short peptides derived from the C-terminal BAG domain, which mediates binding with the ATPase domain of HSC70/HSP70, have been shown to inhibit the interaction between BAG-1 and the heat shock proteins^20^. Inhibition of BAG-1-mediated protein-protein interactions through disruption of BAG-1 binding to HSC70/HSP70 by Thioflavin-S and the C-terminal BAG domain-derived short peptides therefore offers an effective strategy to elucidate the role of BAG-1 in E2/ER-facilitated BMP-directed osteoblast differentiation.

The present study aims to examine the effect of *Bag-1* haploinsufficiency on osteoblast development by analysing the differences in BMP-2-directed osteogenic differentiation of BMSCs of *Bag-1* heterozygous and wild-type mice. Furthermore, the study seeks to investigate the effect of inhibition of the interaction between BAG-1 and HSC70 on E2/ER-facilitated BMP-2-directed osteogenic differentiation of BMSCs.

## Results

### Characterisation of the effects of *Bag-1* haploinsufficiency on proliferation, osteogenic differen-tiation and apoptosis of BMSCs

**Figure 1:**
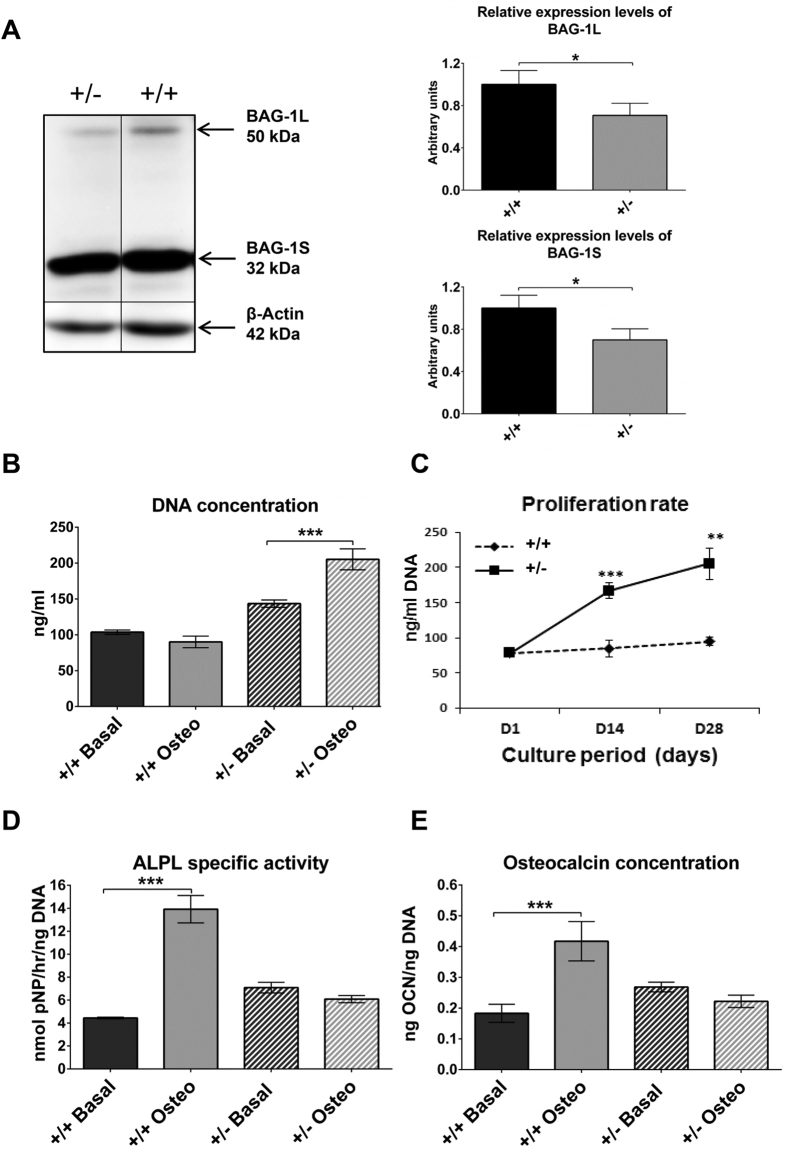
Proliferation and BMP-2-stimulated osteogenic differentiation of BMSCs of 14-week-old *Bag-1*^+/+^ and *Bag-1*^+/−^ female mice. (**A**) Representative immunoblots demonstrating bands for BAG-1L, BAG-1S and β-Actin in day-28 cultures of BMSCs of *Bag-1*^+/−^ and *Bag-1*^+/+^ female mice in basal medium. Densitometric quantification of the bands was performed to measure the expression of the BAG-1L and BAG-1S proteins, data was normalised to β-Actin and plotted in the form of bar graphs. (**B**) DNA concentrations of day-28 cultures of BMSCs of *Bag-1*^+/+^ and *Bag-1*^+/−^ female mice in basal and osteogenic media. (**C**) Cell proliferation profiles over the course of 28-day osteogenic cultures of BMSCs of *Bag-1*^+/+^ and *Bag-1*^+/−^ female mice. For statistical analyses, DNA concentrations were compared between days 1 and 14 of culture, and days 14 and 28 of culture. (**D**) ALPL specific activity and (**E**) osteocalcin concentration were measured in day-28 cultures of BMSCs of *Bag-1*^+/+^ and *Bag-1*^+/−^ female mice in basal and osteogenic media. Results presented as mean ± SD; n = 3 cultures per group; ***P < 0.001, **P < 0.01, *P < 0.05.

Genotyping was performed for the identification of wild-type (+/+), *Bag-1* heterozygous (+/−) and *Bag-1* null (−/−) mice (Supplementary Fig. 1A). The results of genotyping were confirmed by immunoblotting and quantitation of the expression of BAG-1 isoforms in wild-type, heterozygous and null littermates (Supplementary Fig. 1B,C). BMSC cultures of 14-week-old wild-type and *Bag-1* heterozygous mice were utilised for analyses of cell proliferation, osteogenic differentiation and apoptosis. Day-28 cultures in basal medium were harvested for the preparation of cell lysates for immunoblotting. Immunoblots probed with the anti-BAG-1 antibody exhibited bands specific for BAG-1L (50 kDa) and BAG-1S (32 kDa) proteins (Fig. 1A and Supplementary Fig. 2A for female and male mice, respectively). Densitometric quantification of the bands was performed to measure the expression levels of the BAG-1L and BAG-1S proteins. Lower levels of BAG-1L and BAG-1S proteins were observed in BMSC cultures of *Bag-1* heterozygous mice compared to wild-type mice (Fig. 1A and Supplementary Fig. 2A for female and male mice, respectively). The immunoblots also demonstrated intense bands for BAG-1S compared to BAG-1L in BMSC cultures of both wild-type and *Bag-*1 heterozygous mice, indicating higher expression of BAG-1S in comparison to BAG-1L in murine BMSCs.

Due to the direct correlation between cell number and DNA content, cell numbers in day-28 cultures of BMSCs in basal and osteogenic media were determined by measuring the cellular DNA concentration. In wild-type female mice, no differences in DNA concentration (and therefore cell number) were observed between day-28 BMSC cultures in basal and osteogenic media (Fig. 1B). In BMSC cultures of *Bag-1* heterozygous female mice, DNA concentration of day-28 cultures in osteogenic medium was significantly higher than DNA concentration of day-28 cultures in basal medium (Fig. 1B). In both wild-type and *Bag-1* heterozygous male mice, DNA concentrations of day-28 BMSC cultures were significantly lower in osteogenic conditions compared to basal conditions (Supplementary Fig. 2B). In osteogenic medium, BMSCs of wild-type female mice demonstrated negligible rates of proliferation over the 28-day culture period, while BMSCs of *Bag-1* heterozygous female mice exhibited significantly higher rates of proliferation over 28 days (Fig. 1C). BMSCs of wild-type and heterozygous male mice proliferated at significantly higher rates between days 1 and 14 of culture, while proliferation of BMSCs in the two groups of male mice decreased significantly between days 14 and 28 of culture in osteogenic conditions (Supplementary Fig. 2C).

To examine the effect of haploinsufficiency of *Bag-1* on the ability of BMSCs to differentiate into osteoblasts in response to BMP-2, 28-day cultures of BMSCs of wild-type and *Bag-*1 heterozygous mice were analysed for the expression of osteoblast-specific markers, namely Alkaline phosphatase, tissue non-specific isozyme (ALPL) and Osteocalcin. In wild-type female mice, specific activity of ALPL (Fig. 1D) and the concentration of osteocalcin (Fig. 1E) were significantly higher in day-28 BMSC cultures in osteogenic conditions compared to basal conditions. In *Bag-1* heterozygous female mice, no significant differences in ALPL specific activity (Fig. 1D) and osteocalcin concentration (Fig. 1E) were observed between day-28 BMSC cultures in basal and osteogenic media. In contrast, in day-28 cultures of BMSCs of wild-type and *Bag-1* heterozygous male mice, ALPL specific activity (Supplementary Fig. 2D) and osteocalcin concentration (Supplementary Fig. 2E) were significantly higher in osteogenic conditions compared to basal conditions. In comparison to day-28 osteogenic cultures of BMSCs of *Bag-1* heterozygous female mice, day-28 osteogenic cultures of BMSCs of wild-type female mice exhibited higher number of colonies stained with Alizarin red (Supplementary Fig. 3).

Overall, a very low incidence of apoptosis was observed in day-28 BMSC cultures. No differences in cell apoptosis were observed between day-28 basal and osteogenic cultures of BMSCs of wild-type and *Bag-1* heterozygous female (Supplementary Fig. 4A) and male (Supplementary Fig. 4B) mice.

### Differential expression of skeletal development genes between osteogenic cultures of BMSCs of wild-type and *Bag-1* heterozygous mice

The mouse osteogenesis RT^2^ Profiler™ PCR arrays were employed to examine the differences in the expression of 84 genes crucial for skeletal development between day-28 osteogenic cultures of BMSCs of 14-week-old wild-type and *Bag-1* heterozygous female mice. From the 84 genes constituting the array, 30 genes were differentially expressed between day-28 BMSC cultures of *Bag-1*^+/+^ and *Bag-1*^+/−^ female mice in response to the osteogenic stimulus provided by BMP-2. Genes that were upregulated and downregulated in day-28 osteogenic cultures of *Bag-1*^+/−^ BMSCs relative to day-28 osteogenic cultures of *Bag-1*^+/+^ BMSCs, along with the values for fold up/downregulation and statistical significance have been listed (Supplementary Fig. 5A). A volcano plot, constructed by plotting the negative log of the p-value on the y-axis and log of the fold change between the two conditions on the x-axis, enabled rapid identification of the 30 differentially expressed genes (Supplementary Fig. 5B; red circles represent upregulated genes and green circles represent downregulated genes).

**Figure 2:**
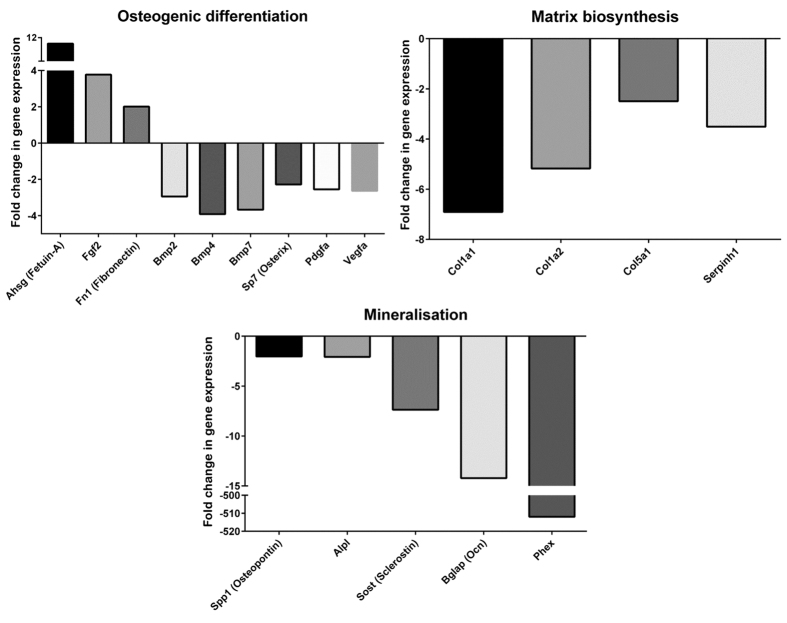
Fold changes in the expression of candidate genes from the mouse osteogenesis RT^2^ Profiler™ PCR array in day-28 osteogenic cultures of BMSCs of 14-week-old *Bag-1*^+/−^ female mice relative to day-28 osteogenic cultures of BMSCs of age-matched *Bag-1*^+^/^+^ female mice. Fold changes in the expression of genes crucial for osteogenic differentiation, bone matrix synthesis and mineralization were plotted in the form of bar graphs. The PCR arrays were performed in triplicate and the results were analysed using RT^2^Profiler PCR array data analysis version 3.5. The threshold for the fold–change in gene expression was set at 2 and the significance of the difference in gene expression was calculated with the P-value set at 0.05.

Nine genes were significantly upregulated in day-28 osteogenic cultures of *Bag-1*^+/−^ BMSCs compared to day-28 osteogenic cultures of *Bag-1*^+/+^ BMSCs. The upregulated genes included *Ahsg, Fgf-2, Fn-1*, which have important roles in regulation of osteogenic differentiation, *Col14a1, Col4a1, Gdf-10, Mmp-8, Tnfsf-11* and *Vcam-1*, which have important functions in collagen fibril organisation, remodelling of the extracellular matrix, osteoclastogenesis and cell adhesion (Fig. 2 and Supplementary Fig. 5A). Expression levels of 21 genes were downregulated in day-28 osteogenic cultures of *Bag-1*^+/−^ BMSCs, in comparison to day-28 osteogenic cultures of *Bag-1*^+/+^ BMSCs. Key genes whose expression levels were downregulated included *Bmp-2, Bmp-4, Bmp-7, Sp7, Pdgfa, Vegfa*, which have important roles in regulation of osteogenic differentiation, *Col1a1, Col1a2, Col5a1, Serpinh-1*, which are important for collagen biosynthesis and whose products are vital constituents of the extracellular matrix, *Spp1, Alpl, Sost, Bglap* and *Phex*, which have regulatory roles in bone matrix mineralisation (Fig. 2).

### Effect of estrogen on BMP-2-directed osteogenic differentiation of BMSCs

The expression levels of genes encoding the estrogen receptors, namely *ERα* and *ERβ*, in day-28 cultures (in basal medium) of BMSCs of 14-week-old wild-type mice were compared to murine osteoblast-like MC3T3-E1 cells, which have been shown to express both receptor subtypes^30^. Mouse BMSCs were found to express genes encoding the two ER isoforms; in comparison to MC3T3-E1 cells, expression of *ERα* was significantly lower in BMSCs, while expression of *ERβ* was significantly higher in BMSCs (Supplementary Fig. 6A). Osteogenic culture medium was supplemented with 17-β-estradiol (E2) in concentrations of 10 pM, 100 pM, 10 nM and 100 nM to determine the optimal concentration of E2 capable of promoting BMP-2-directed osteogenic differentiation of BMSCs. Following culture for 28 days in basal, osteogenic and osteogenic media supplemented with different concentrations of E2, expression of *Alpl* was determined as an early marker of osteogenic differentiation of BMSCs. Expression of *Alpl* in wild-type BMSCs was significantly upregulated in response to BMP-2 in the osteogenic medium compared to basal culture conditions (Supplementary Fig. 6B). Moreover, in comparison to BMSCs cultured in osteogenic medium, statistically significant increase in the expression of *Alpl* was observed in BMSCs cultured for 28 days in osteogenic medium supplemented with 100 nM E2 (Supplementary Fig. 6B). Furthermore, culture of BMSCs of 14-week-old wild-type and *Bag-1* heterozygous female mice for 28 days in osteogenic medium supplemented with 100 nM E2 resulted in significant increases in the expression of *Bmpr2* (gene encoding BMP receptor Type II), compared to BMSC cultures in osteogenic medium (Supplementary Fig. 6C).

### Analysis of inhibition of the interaction between BAG-1 and HSC70 by Thioflavin-S on E2/ER-facilitated BMP-2-directed osteogenic differentiation of BMSCs

Thioflavin-S, a small-molecule chemical inhibitor of the interaction between BAG-1 and heat shock proteins, was used to investigate the functional significance of BAG-1-mediated protein-protein interactions, specifically, BAG-1-regulated activation of ER by HSC70, in E2-facilitated BMP-2-directed osteogenic differentiation of BMSCs. BMSCs of 14-week-old *Bag-1*^+/+^ and *Bag-1*^+/−^ female mice were cultured for 28 days in basal medium, osteogenic medium, osteogenic medium supplemented with 100 nM E2 and osteogenic medium supplemented with 100 nM E2 and 5 μM Thioflavin-S. Day-28 BMSC cultures were harvested for the analyses of expression of osteogenic genes, namely *Runx-2, Osterix, Alpl* and *Osteocalcin*, estimation of ALPL specific activity and quantitation of DNA concentration.

**Figure 3:**
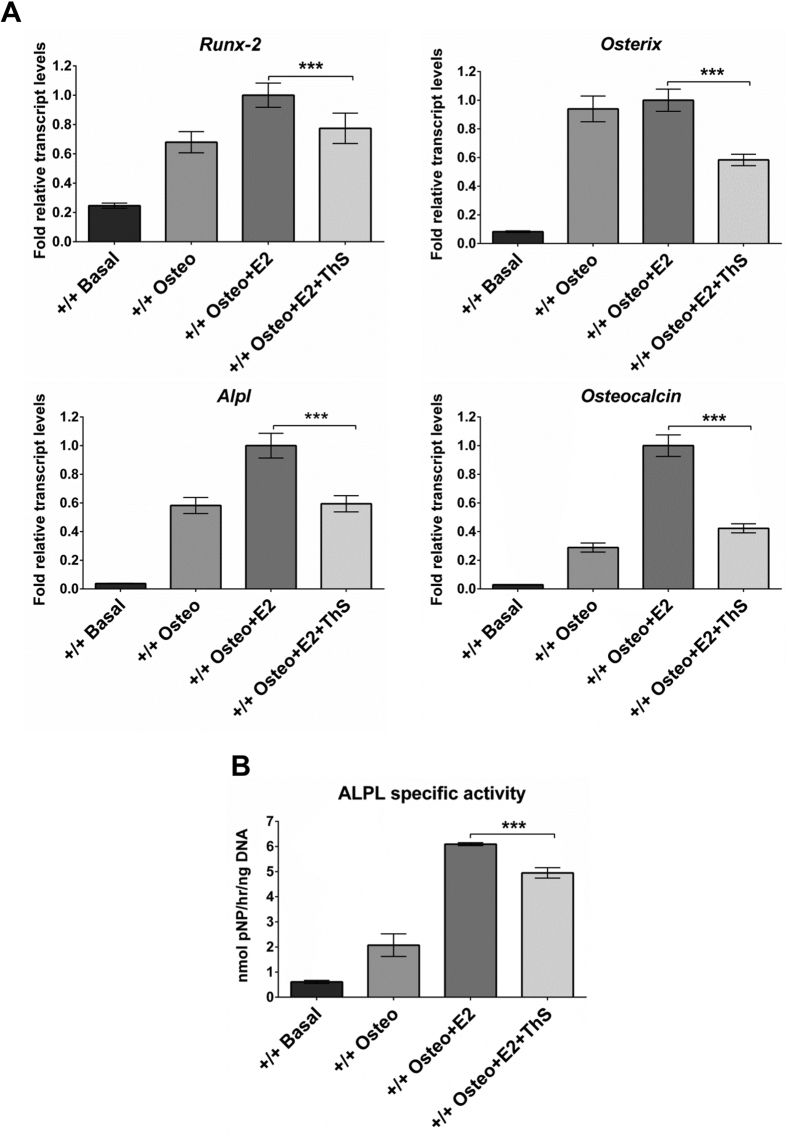
Effect of Thioflavin-S (ThS), a small-molecule chemical inhibitor of the interaction between BAG-1 and HSC70, on E2/ER-facilitated BMP-2-directed osteogenic differentiation of BMSCs of 14-week-old *Bag-1*^+^/^+^ female mice. (**A**) Expression levels of osteogenic genes, namely *Runx-2, Osterix, Alpl* and *Osteocalcin*, were measured in day-28 cultures of BMSCs in basal, osteogenic, osteogenic + E2, osteogenic + E2 + Thioflavin-S media. Fold changes (2^−ΔΔC^_T_) in relative mRNA transcript levels were normalised to the endogenous reference (β-Actin) and plotted as bar graphs. The range of gene expression was indicated by 2^−(ΔΔC^_T_^+ SD)^ and 2^−(ΔΔC^_T_^−SD)^, where SD: standard deviation of the ΔΔC_T_/ΔC_T_ value. (**B**) Specific activity of ALPL (presented as mean ± SD) was measured in day-28 cultures of BMSCs in basal, osteogenic, osteogenic + E2, osteogenic + E2 + Thioflavin-S media; n = 3 cultures per group; ***P < 0.001.

**Figure 4:**
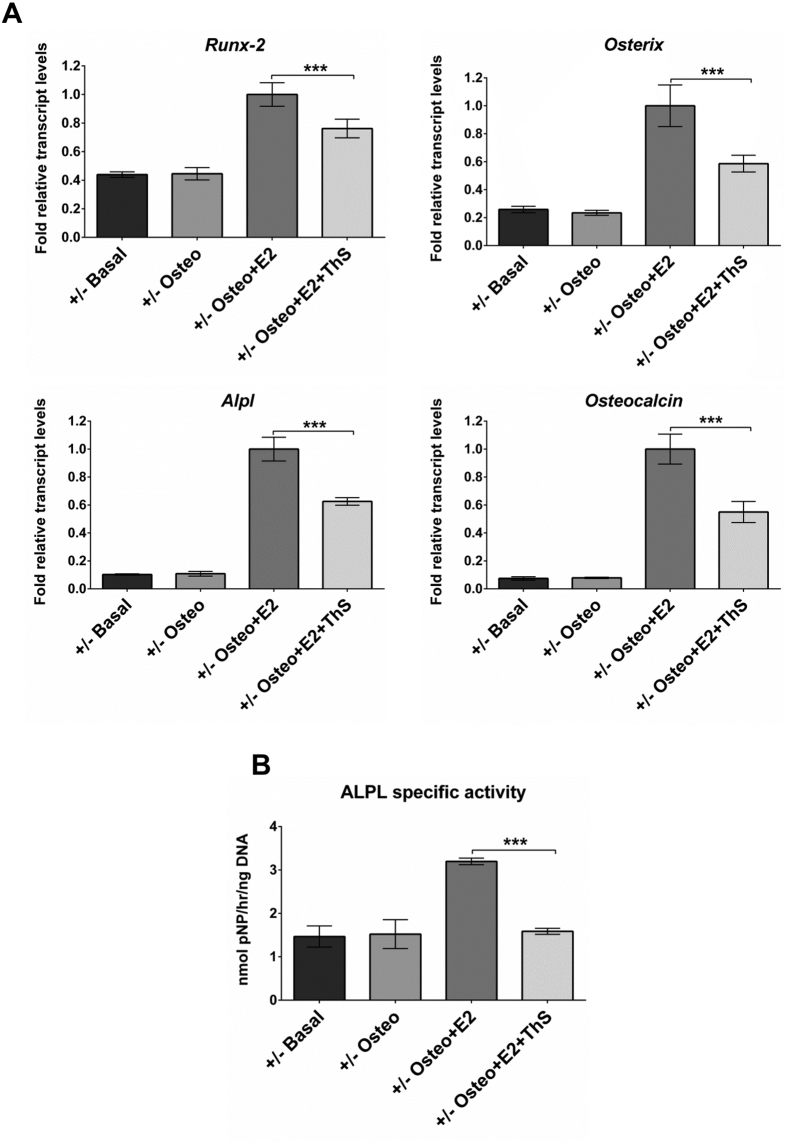
Effect of Thioflavin-S (ThS), a small-molecule chemical inhibitor of the interaction between BAG-1 and HSC70, on E2/ER-facilitated BMP-2-directed osteogenic differentiation of BMSCs of 14-week-old *Bag-1*^+/−^ female mice. (**A**) Expression levels of osteogenic genes, namely *Runx-2, Osterix, Alpl* and *Osteocalcin*, were measured in day-28 cultures of BMSCs in basal, osteogenic, osteogenic + E2, osteogenic + E2 + Thioflavin-S media. Fold changes (2^−ΔΔC^_T_) in relative mRNA transcript levels were normalised to the endogenous reference (β-Actin) and plotted as bar graphs. The range of gene expression was indicated by 2^−(ΔΔC^_T_^+SD)^ and 2^−(ΔΔC^_T_^−SD)^, where SD: standard deviation of the ΔΔC_T_/ΔC_T_ value. (**B**) Specific activity of ALPL (presented as mean ± SD) was measured in day-28 cultures of BMSCs in basal, osteogenic, osteogenic + E2, osteogenic + E2 + Thioflavin-S media; n = 3 cultures per group; ***P < 0.001.

Compared to cultures in basal medium, day-28 cultures of BMSCs of *Bag-1*^+/+^ mice exhibited significantly increased expression of osteogenic genes (Fig. 3A) and significantly higher specific activity of ALPL (Fig. 3B) in osteogenic and E2-supplemented osteogenic media. Moreover, the osteogenic response was higher in day-28 cultures of BMSCs of *Bag-1*^+/+^ mice in osteogenic medium supplemented with E2, in comparison to day-28 cultures in osteogenic medium. BMSCs of *Bag-1*^+/−^ female mice did not exhibit robust osteogenic differentiation in day-28 cultures in osteogenic medium compared to cultures in basal medium (Fig. 4A,B). This observation was in keeping with our previous findings, which demonstrated decreased osteogenic response to BMP-2 by BMSCs of *Bag-1*^+/−^ female mice (Fig. 1D,E). Remarkably, expression of osteogenic genes (Fig. 4A) and specific activity of ALPL (Fig. 4B) were significantly upregulated in day-28 BMSC cultures of *Bag-1*^+/−^ female mice in osteogenic medium supplemented with E2, compared to day-28 cultures of *Bag-1*^+/−^ BMSCs in osteogenic and basal media. In comparison to the robust osteogenic differentiation of *Bag-1*^+/+^ and *Bag-1*^+/−^ BMSCs in day-28 cultures in E2-supplemented osteogenic medium, expression of osteogenic genes (Figs 3A and 4A) and specific activity of ALPL (Figs 3B and 4B) were significantly decreased in day-28 cultures of BMSCs of *Bag-1*^+/+^ and *Bag-1*^+/−^ mice in E2-containing osteogenic medium supplemented with Thioflavin-S.

### Effect of inhibition of binding of BAG-1 to HSC70 by the BAG domain-derived short peptide on E2/ER-facilitated BMP-2-directed osteogenic differentiation of BMSCs

BAG-1 binds to the ATPase domain of HSC70 via a very small region comprising of 8 amino acid residues in helix 2 of the C-terminal BAG domain. A short peptide, H2-Penetratin, containing the 8 amino acid binding core of helix 2 of the BAG domain coupled via a peptide bond to Penetratin, was synthesised to inhibit the binding of BAG-1 to HSC70. A mutant peptide, H2mutant-Penetratin, with alanine substitutions for arginine residues at positions 205 and 206 that represented the ‘hot-spot’ on the interaction surface between BAG-1 and HSC70, was also synthesized as the control peptide. BMSCs of 14-week-old *Bag-1*^+/+^ and *Bag-1*^+/−^ female mice were cultured for 28 days in basal medium, osteogenic medium supplemented with 100 nM E2 and osteogenic medium supplemented with 100 nM E2 in addition to either 10 μM H2-Penetratin or 10 μM H2mutant-Penetratin. Day-28 BMSC cultures were harvested for the analyses of expression of osteogenic genes, namely *Runx-2, Osterix, Alpl* and *Osteocalcin*, and estimation of ALPL specific activity, osteocalcin and DNA concentrations.

**Figure 5:**
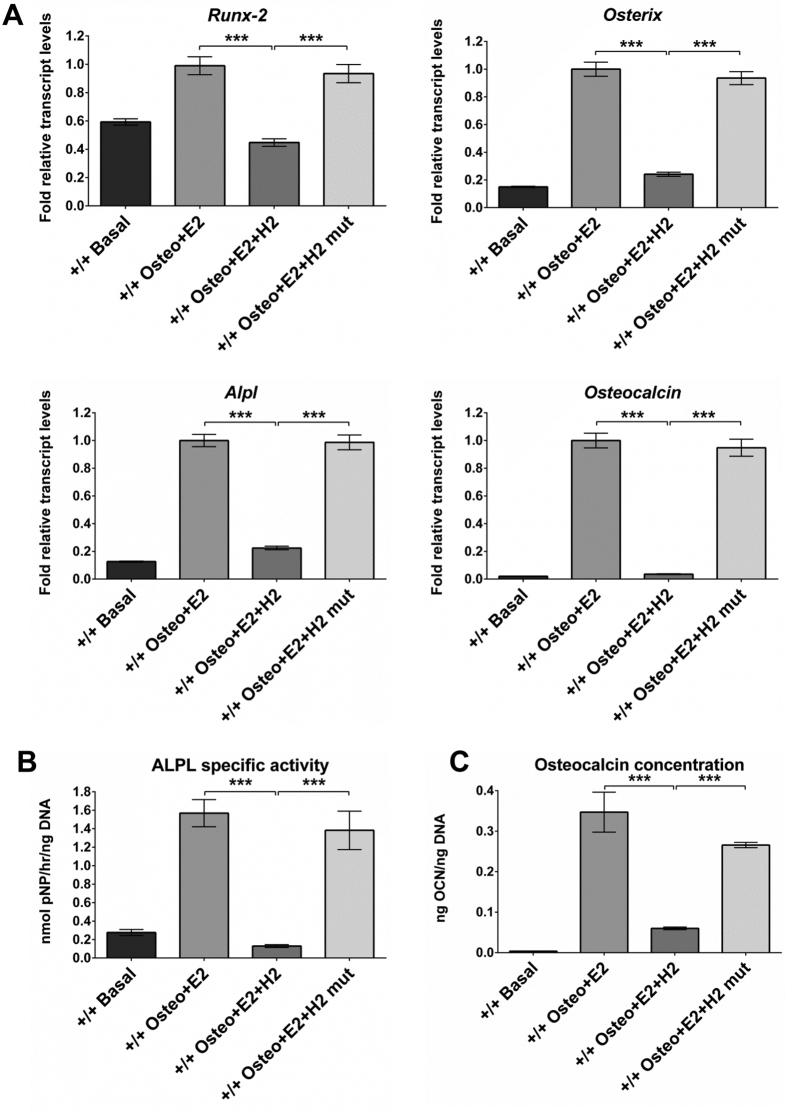
Effect of inhibition of BAG-1 binding to HSC70 by the C-terminal BAG domain-derived short peptide (H2-Penetratin) on E2/ER-facilitated BMP-2-directed osteogenic differentiation of BMSCs of 14-week-old *Bag-1*^+^/^+^ female mice. (**A**) Expression levels of osteogenic genes, namely *Runx-2, Osterix, Alpl* and *Osteocalcin*, were measured in day-28 cultures of BMSCs in basal, osteogenic + E2, osteogenic + E2 + H2-Penetratin, osteogenic + E2 + H2mutant-Penetratin media. Fold changes (2^−ΔΔC^_T_) in relative mRNA transcript levels were normalised to the endogenous reference (β-Actin) and plotted as bar graphs. The range of gene expression was indicated by 2^−(ΔΔC^_T_^+SD)^ and 2^−(ΔΔC^_T_^−SD)^, where SD: standard deviation of the ΔΔC_T_/ΔC_T_ value. (**B**) Specific activity of ALPL and (**C**) concentration of osteocalcin were determined in day-28 cultures of BMSCs in basal, osteogenic + E2, osteogenic + E2 + H2-Penetratin, osteogenic + E2 + H2mutant-Penetratin media. Results for ALPL specific activity and osteocalcin concentration presented as mean ± SD; n = 3 cultures per group; ***P < 0.001.

**Figure 6:**
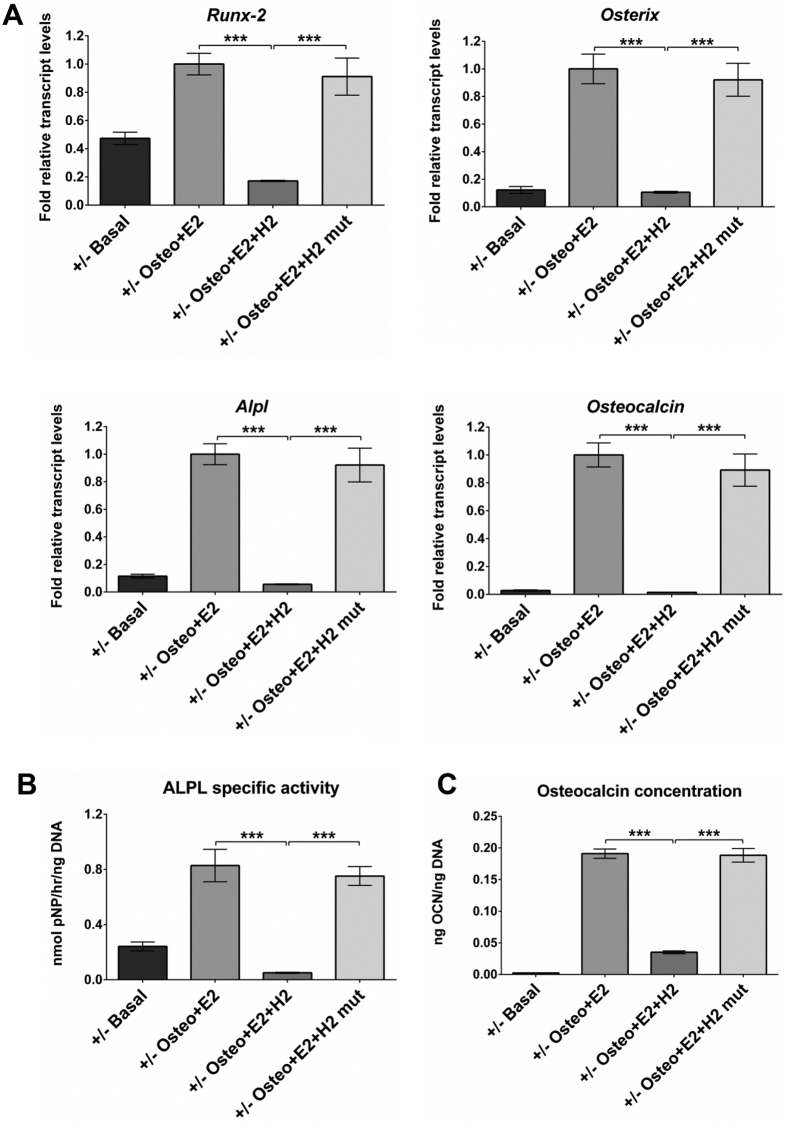
Effect of inhibition of BAG-1 binding to HSC70 by the C-terminal BAG domain-derived short peptide (H2-Penetratin) on E2/ER-facilitated BMP-2-directed osteogenic differentiation of BMSCs of 14-week-old *Bag-1*^+/−^ female mice. (**A**) Expression levels of osteogenic genes, namely *Runx-2, Osterix, Alpl* and *Osteocalcin*, were measured in day-28 cultures of BMSCs in basal, osteogenic + E2, osteogenic + E2 + H2-Penetratin, osteogenic + E2 + H2mutant-Penetratin media. Fold changes (2^−ΔΔC^_T_) in relative mRNA transcript levels were normalised to the endogenous reference (β-Actin) and plotted as bar graphs. The range of gene expression was indicated by 2^−(ΔΔC^_T_^+ SD)^ and 2^−(ΔΔC^_T_^−SD)^, where SD: standard deviation of the ΔΔC_T_/ΔC_T_ value. (**B**) Specific activity of ALPL and (**C**) concentration of osteocalcin were determined in day-28 cultures of BMSCs in basal, osteogenic + E2, osteogenic + E2 + H2-Penetratin, osteogenic + E2 + H2mutant-Penetratin media. Results for ALPL specific activity and osteocalcin concentration presented as mean ± SD; n = 3 cultures per group; ***P < 0.001.

In comparison to day-28 cultures in basal medium, significant increases in the expression of osteogenic genes (Figs 5A and 6A), specific activity of ALPL (Figs 5B and 6B) and osteocalcin concentration (Figs 5C and 6C) were observed in day-28 cultures of BMSCs of *Bag-1*^+/+^ and *Bag-1*^+/−^ mice in osteogenic medium supplemented with E2. Significant decreases in the expression of osteogenic genes (Figs 5A and 6A), specific activity of ALPL (Figs 5B and 6B) and osteocalcin concentration (Figs 5C and 6C) were observed in day-28 cultures of BMSCs of *Bag-1*^+/+^ and *Bag-1*^+/−^ mice in osteogenic medium supplemented with E2 and H2-Penetratin, compared to day-28 cultures in osteogenic medium supplemented with E2. In contrast, H2mutant-Penetratin had no effect on E2/ER-facilitated BMP-2-directed osteogenic responses of BMSCs of *Bag-1*^+/+^ and *Bag-1*^+/−^ mice. This was demonstrated by no differences in the expression of osteogenic genes (Figs 5A and 6A), specific activity of ALPL (Figs 5B and 6B) and osteocalcin concentration (Figs 5C and 6C) between day-28 BMSC cultures in E2-supplemented osteogenic medium and osteogenic medium supplemented with E2 and H2mutant-Penetratin. Moreover, expression of osteogenic genes (Figs 5A and 6A), specific activity of ALPL (Figs 5B and 6B) and osteocalcin concentration (Figs 5C and 6C) were significantly higher in day-28 BMSC cultures in osteogenic medium supplemented with E2 and H2mutant-Penetratin, compared to day-28 cultures in osteogenic medium supplemented with E2 and H2-Penetratin.

### Effects of Thioflavin-S and the BAG-domain-derived short peptides on BMSC viability and proliferation

**Figure 7:**
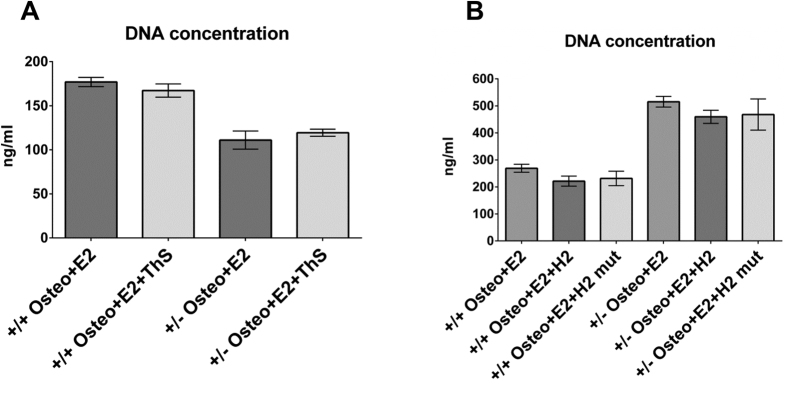
DNA concentrations of day-28 cultures of BMSCs of 14-week-old *Bag-1*^+^/^+^ and *Bag-1*^+/−^ female mice. The DNA concentrations of day-28 cultures of BMSCs in osteogenic media supplemented with E2 were not altered by the addition of Thioflavin-S (**A**) and C-terminal BAG domain-derived short peptides (**B**). Results presented as mean ± SD; n = 3 cultures per group.

No differences in cell number/DNA content between day-28 cultures of BMSCs (of both *Bag-1*^+/+^ and *Bag-1*^+/−^ mice) in E2-supplemented osteogenic medium and osteogenic medium supplemented with E2 and Thioflavin-S confirmed that Thioflavin-S was not toxic to the BMSCs (Fig. 7A). Similarly, BMSC viability and proliferation were unaffected by the short peptides, namely H2-Penetratin and H2mutant-Penetratin, as demonstrated by no differences in cell number/DNA concentrations between day-28 cultures of BMSCs (of both *Bag-1*^+/+^ and *Bag-1*^+/−^ mice) in E2-supplemented osteogenic medium and osteogenic medium supplemented with E2 and the short peptides (Fig. 7B).

## Discussion

The present study has highlighted the significance of BAG-1-regulated activation of ER by HSC70 in the modulation of E2-facilitated BMP-2-stimulated osteogenic differentiation of murine BMSCs. This work has therefore demonstrated for the first time the importance of BAG-1-mediated protein-protein interactions in the regulation of osteoblast development. While both BAG-1 isoforms were expressed by murine BMSCs, expression of BAG-1S was observed to be higher than BAG-1L. This finding is in keeping with observations made by previous studies that BAG-1S is the major BAG-1 isoform in rodent cells^11^,^15^.

To elucidate the causes of decreased BMP-2-directed osteogenic differentiation of BMSCs of *Bag-1*^+/−^ female mice, differences in the expression of an array of genes crucial for skeletal development were analysed between osteogenic cultures of BMSCs of *Bag-1*^+/−^ and *Bag-1*^+/+^ female mice. Increased expression of *Ahsg* (Fetuin), *Fgf2* (Fibroblast growth factor-2) and *Fn1* (Fibronectin) in osteogenic cultures of BMSCs of *Bag-1*^+/−^ female mice contributed, in part, to the lower osteogenic response of the cells to BMP-2 and the increased rate of cell proliferation. Fetuin acts as an inhibitor of osteogenesis in rodent BMSC cultures due to its high affinity for binding to BMP2/4/6, thereby resulting in the inhibition of the osteogenic activity of BMPs^31^. Similarly, FGF-2 is shown to inhibit BMP-stimulated osteoblast differentiation and increased levels of FGF-2 are found to maintain osteoprogenitor cells in a proliferative state by delaying their subsequent differentiation, maturation and mineralisation^32^,^33^. Fibronectin, an extracellular matrix glycoprotein, is synthesised by osteoblasts and accumulates in the extracellular matrix during cell proliferation and early differentiation, while synthesis of fibronectin is dramatically reduced with cell maturation^34^. It is therefore possible that significant upregulation of *Fn1* expression in osteogenic cultures of BMSCs of *Bag-1*^+/−^ female mice, in comparison to osteogenic cultures of BMSCs of *Bag-1*^+/+^ female mice, reflects the highly proliferative/early differentiation state of the cells.

The important roles of BMP-2, BMP-4 and BMP-7 in the induction of differentiation of osteoblast lineage cells have been widely recognised^35^,^36^,^37^. Downregulation of expression of the osteoinductive BMPs could have contributed to decreased osteogenic differentiation of *Bag-1*^+/−^ BMSCs in comparison to *Bag-1*^+/+^ BMSCs. Additionally, expression levels of *SP7* (encoding the transcription factor Osterix), crucial for differentiation of preosteoblasts into functional osteoblasts^38^, *Pdgfa*, capable of regulating osteogenesis via autocrine and paracrine effects^39^ and *Vegfa*, responsible for promoting bone formation through induction of neovascularisation or by directly stimulating osteoblast differentiation^40^, were significantly downregulated in osteogenic cultures of *Bag-1*^+/−^ BMSCs.

Furthermore, downregulation of expression of *Col1a1, Col1a2, Col5a1, Serpinh1, Alpl, Spp1, Bglap* and *Phex* in osteogenic cultures of BMSCs of *Bag-1*^+/−^ female mice contributed to decreased synthesis of the bone matrix and its mineralisation. Type I collagen is the most abundant collagen in bone matrix, while Type V collagen is essential for the formation of normal Type I collagen fibrils due to the critical role of Type V collagen in early fibril initiation, determination of fibril structure and matrix organisation^41^. The collagen-specific molecular chaperone, Serpinh-1/HSP47, is located in the endoplasmic reticulum and plays an important role in collagen biosynthesis^42^. Matrix mineralisation is regulated by ALPL, an enzyme with a pivotal role in mineralisation^43^, Osteopontin (*Spp1*), which has key roles in mineral-binding and regulation of crystal growth^44^, Osteocalcin (*Bglap*), an osteoblast-specific protein characterised by the presence of γ-carboxyglutamic acid (Gla) residues, which has a regulatory role in bone mineral maturation^45^,^46^ and *Phex* (Phosphate-regulating gene with homologies to endopeptidases on the X chromosome), encoding a protease responsible for the degradation and clearance of mineralisation-inhibiting ASARM (acidic serine and aspirate-rich motif) peptides that are derived from a group of extracellular matrix SIBLING (small integrin-binding ligand N-linked glycoprotein) proteins including MEPE (matrix extracellular phosphoglycoprotein) and osteopontin^47^.

Interestingly, expression of *Tnsf11* was upregulated almost 4-fold in BMSC cultures of *Bag-1*^+/−^ female mice compared to BMSC cultures of *Bag-1*^+/+^ female mice. The cytokine TNFSF11 (Tumor necrosis factor (ligand) superfamily, member 11 or RANKL: Receptor activator of nuclear factor kappa-B ligand), alongside macrophage colony stimulating factor (M-CSF), is essential for osteoclast formation^48^. Interaction of RANKL, expressed primarily on the surface of stromal cells and osteoblasts in the bone marrow, with RANK, expressed on the surface of blood-derived mononuclear osteoclast precursors, stimulates osteoclastogenesis^49^. Co-cultures of cells harvested from day-28 osteogenic cultures of BMSCs of *Bag-1*^+/−^ female mice with blood-derived mononuclear cells will be established, as part of the future work, to determine whether osteoclastogenesis of the monocytic osteoclast precursors is enhanced in response to increased expression of RANKL by the BMSCs.

*Bag-1* haploinsufficiency did not affect the osteogenic response of BMSCs of *Bag-1* heterozygous male mice to BMP-2; this highlighted the need to understand the role of interacting factors capable of modulating gender differences in BMP-2-directed osteogenic differentiation of BMSCs of *Bag-1*^+/−^ mice. Estrogen, the primary female sex hormone, binds to estrogen receptors, ligand-activated enhancer proteins that are members of the NHR superfamily, and is functionally involved in the process of BMP-directed osteoblast differentiation of osteoprogenitor cells via upregulation of *Bmpr2* expression and Smad 1/5/8 phosphorylation^28^. The present study has demonstrated the expression of ERα and ERβ by primary murine BMSCs and significant upregulation of *Bmpr2* expression in BMP-2-treated cultures of BMSCs supplemented with 100 nM E2. Co-treatment of mouse BMSCs with BMP-2 and E2 has been shown to enhance osteogenic lineage commitment of the multipotent cell population at the expense of adipogenic differentiation and the modulation of BMSC lineage shift towards the osteoblast phenotype by E2 occurred in an ER-specific manner, but without ER subtype specificity^27^. Thus, E2 increased the responsiveness of ER-expressing BMSCs of *Bag-1*^+/+^ and *Bag-1*^+/−^ female mice to BMP-2 through upregulation of the expression of *Bmpr2* and facilitated robust osteogenesis by enhancing the commitment of the BMSC population to differentiate into the osteoblast lineage.

Thioflavin S functions as a small molecule chemical inhibitor of the interaction between BAG-1 and HSC70, since the binding site for the compound lies within helix 2 of the BAG domain; alternatively, it is possible that Thioflavin S may bind elsewhere within BAG-1 and elicit a conformational change in the BAG domain, leading to the loss of multiple interaction partners, including HSC70^29^. Through its interaction with HSC70, BAG-1 can regulate the activation of NHRs, including ERs, and play an important role in the modulation of cellular responses to steroid hormones, such as E2^16^,^17^. In the present study, Thioflavin S disrupted the binding between HSC70 and BAG-1 in BMSC cultures supplemented with BMP-2 and E2, resulting in the downregulation of E2-facilitated BMP-2-directed osteogenic differentiation of BMSCs. Thioflavin S therefore served as a useful tool to elucidate the functional significance of BAG-1-regulated activation of ER by HSC70 in E2-facilitated BMP-2-directed osteogenic differentiation of BMSCs. However, Thioflavin S/NSC71948 is a mixture of components arising from the methylation and sulfonation of primulin base, the selectivity profile of which is not fully characterised and thus, the likelihood of off-target effects cannot be ruled out^50^,^51^.

Hence, to improve selectivity/specificity, a short peptide, H2-Penetratin, containing the 8 amino acid binding core of helix 2, coupled via a peptide bond at its C-terminus to Penetratin, a fragment of the Antennaepaedia protein that enhances cellular uptake, was used to inhibit the binding between BAG-1 and HSC70 in BMSC cultures supplemented with BMP-2 and E2. The H2-Penetratin peptide has been demonstrated to bind the ATPase domain of HSC70 and exert its effects, such as growth inhibition, in human breast cancer cells by preventing the interaction between BAG-1 and HSC70^20^. Similarly, in our study, inhibition of binding of BAG-1 to HSC70 by H2-Penetratin resulted in significant downregulation of E2/ER-facilitated BMP-2-directed osteogenic responses of BMSCs of both *Bag-1*^+/+^ and *Bag-1*^+/−^ mice. The mutant peptide, with alanine substitutions for arginine residues at positions 205 and 206 representing the ‘hot-spot’ on the interaction surface between BAG-1 and HSC70, was significantly less effective in downregulating the osteogenic responses of the BMSCs, thereby confirming the specificity of the H2-Penetratin peptide to disrupt the interaction between BAG-1 and HSC70 in BMSCs and cause downregulation of E2/ER-facilitated BMP-2-directed osteogenic differentiation.

Since no differences were observed in cell number/DNA content between day-28 cultures of BMSCs in osteogenic medium supplemented with E2 and osteogenic medium supplemented with E2, Thioflavin-S or the BAG domain-derived short peptides, decreased E2/ER-facilitated BMP-2-directed osteogenic responses of BMSCs in presence of Thioflavin S and the H2-Penetratin peptide were not due to compromised cell viability. It is, however, important to note that, in addition to HSC70 binding, the BAG domain facilitates binding of BAG-1 to RAF-1 through helices 1 and 2^52^. As the binding sites for HSC70 and RAF-1 overlap, which can result in competitive binding, more specific mutations in the HSC70 binding region of the BAG domain will need to be created as part of the future work to gain valuable information regarding the significance of the independent role of the interaction of HSC70 with BAG-1 in the regulation of E2/ER-facilitated BMP-2-directed osteogenic differentiation of BMSCs.

In summary, the present study has demonstrated that deletion of one functional *Bag-1* allele decreased the potential of BMSCs of *Bag-1*^+/−^ female mice to differentiate into osteoblasts in response to BMP-2. Although there are many mechanisms and targets of BAG-1 function, the ability of BAG-1 to regulate the activation of NHRs, critically, through its interaction with the HSC70/HSP70 chaperone proteins, has now been accepted as one of the key mechanisms of regulation of cellular functions by BAG-1. By highlighting the significance of the interaction between BAG-1 and HSC70 in the modulation of E2/ER-facilitated BMP-2-directed osteogenic differentiation of BMSCs, our study has provided valuable insight into the role of BAG-1-mediated protein-protein interactions in the regulation of osteoblast development. Generation of conditional knockout mice in which the *Bag-1* gene is deleted from osteoblast-lineage cells would aid comprehensive investigation of the role of *Bag-1* in bone development.

## Materials and Methods

Chemicals and reagents were purchased from Invitrogen (Paisley, UK) and Sigma-Aldrich (Gillingham, UK) unless specified.

### *Bag-1* ^+/−^ mice

The protocol for targeted inactivation of the mouse *Bag-1* locus and the PCR strategy for genotyping *Bag-1*^−/−^, *Bag-1*^+/−^ and *Bag-1*^+/+^ mice have been described previously^12^,^14^. The *Bag-1*^+/−^ mice were provided by Prof. Stefan Wiese (Ruhr University Bochum, Germany) and bred in the Biomedical Research Facility, University of Southampton. The mice were housed in appropriate environments maintained at 21 °C ± 2 °C and 55% ± 10% humidity with 15–20 room air changes per hour and 12-h/12-h light/dark cycles. The mice had free access to water and were fed *ad libitum*. All animal experimentation was performed under licence (Ref. No. 70/6422) granted by the Home Office (UK) in accordance with the Animals (Scientific Procedures) Act 1986.

Health and Safety risk assessments were performed for all experimental protocols and the protocols were approved by the Research Governance Office, University of Southampton.

### Cultures of mouse BMSCs

Femora and tibiae were dissected from 14-week-old *Bag-1*^+/−^ and *Bag-1*^+/+^ mice for isolation of BMSCs using an established protocol^53^. For each experiment, BMSCs were pooled from the femora and tibiae of 3–4 mice. The cells were plated in 6-well plates at a seeding density of 500,000 cells/cm^2^. After 6 days of culture in basal medium (phenol red-free α-MEM containing 1% penicillin/streptomycin, 10% charcoal stripped fetal calf serum/FCS and 50 μM β-mercaptoethanol), non-adherent red blood cells were removed by washing the cultures using sterile PBS and tissue culture plastic-adherent stromal cells were cultured for further 22 days in basal medium, osteogenic medium (basal medium supplemented with 100 ng/ml rhBMP-2, 100 μM ascorbate-2-phosphate), osteogenic medium supplemented with E2 (10pM, 100pM, 10 nM, 100 nM), osteogenic medium supplemented with E2 (100 nM) and Thioflavin-S (5 μM), osteogenic medium supplemented with E2 (100 nM) and H2-Penetratin/H2mutant-Penetratin (10 μM). As Thioflavin-S was reconstituted in dimethyl sulfoxide (DMSO) and the effective concentration of DMSO in the culture medium containing 5 μM Thioflavin-S was 0.05%, osteogenic medium containing 100 nM E2 was supplemented with 0.05% DMSO in the experiments investigating the effect of Thioflavin-S. Cultures were harvested for detection of apoptosis, biochemical and molecular biology investigations.

The peptides were ordered from ProteoGenix SAS (Schiltigheim, France) and reconstituted in ultrapure water. H2-Penetratin, *CKLDRRVK*ATIE**RQIKIWFQNRRMKWKK**, contained the 8 amino acid binding core of helix 2 (sequence in italics), coupled via a peptide bond at its C-terminus to Penetratin (sequence in bold), a cell permeable fragment of the Antennaepaedia protein that enhanced cellular uptake. H2mutant-Penetratin, *AALDAAVK*ATIE**RQIKIWFQNRRMKWKK**, was characterised by alanine (A) substitutions, particularly for arginine (R) residues at positions 205 and 206 (underlined) that represented the ‘hot-spot’ on the interaction surface between BAG-1 and HSC70.

### Alizarin red staining

Day-28 cultures of BMSCs were fixed using 4% paraformaldehyde. Following a PBS wash, Alizarin red solution (40 mM, pH 4.1–4.3) was added to wells and the plates were incubated at room temperature for 20 minutes on the shaker. Cultures were then washed number of times with fresh ultrapure water to remove the excess stain. Colonies stained with Alizarin red were observed using the Zeiss Axiovert 200 inverted fluorescence microscope (Carl Zeiss, Cambridge, UK) and imaged using a CCD camera with Axiovision imaging software.

### Detection of cell apoptosis

Cell cultures were fixed using 4% paraformaldehyde and terminal deoxynucleotidyl transferase-mediated dUTP nick-end labeling (TUNEL) was performed using the Apoptosis Detection System (Promega, Southampton, UK) according to the manufacturer’s instructions. Positive controls for detection of DNA fragmentation were prepared by treating BMSCs with DNase-1. Nuclei were labelled with Propidium iodide, staining was observed using the Zeiss Axiovert 200 inverted fluorescence microscope and imaged using a CCD camera with Axiovision imaging software. To quantify the results of apoptosis, Propidium iodide-stained nucleated cells and TUNEL-positive apoptotic cells were counted in five different fields of view, and the number of apoptotic cells was calculated as percentage of the total cell number.

### Biochemical investigations

Cells harvested from the cultures were lysed in 0.05% Triton-X and the cell lysates were used for biochemical analyses, namely DNA quantification, estimation of ALPL specific activity and osteocalcin concentration.

### DNA quantification

The fluorescent PicoGreen^®^ double-stranded DNA quantification reagent was used for DNA quantification as described previously^54^. The fluorescence was measured using the FLX-800 microplate fluorescence reader (excitation wavelength of 485 nm, emission wavelength of 530 nm) equipped with the BioTek KC4 KinetiCalc for Windows software (BioTek, Swindon, UK). The DNA content of the sample was quantified by comparison of the sample fluorescence to the fluorescence of a set of standards that were included in every sample run.

### Estimation of ALPL specific activity

ALPL specific activity was measured as previously described^54^. In brief, the method involved the conversion of p-nitrophenyl phosphate by ALP to p-nitrophenol (pNP) and inorganic phosphate under alkaline conditions. The absorbance was measured at 410 nm using ELX-800 universal microplate reader equipped with the BioTek KC4 KinetiCalc for Windows software (BioTek, Swindon, UK). The concentration of pNP was quantified by comparison of the sample absorbance to the absorbance of a set of pNP standards that were included in every sample run. ALPL specific activity was calculated and expressed as nmol pNP/ng DNA/hr.

### Quantification of osteocalcin concentration

Concentration of osteocalcin was measured using the mouse osteocalcin EIA kit (Bioquote Ltd., York, UK), a sandwich ELISA assay, following manufacturer’s instructions. The absorbance was measured at 450 nm using the microplate reader and concentration of osteocalcin was quantified by comparison of the sample absorbance to the absorbance of a set of osteocalcin standards that were included in every sample run. The values were expressed as ng osteocalcin/ng DNA.

### Real-time quantitative PCR

Total RNA was isolated using the RNeasy kit (Qiagen, Manchester, UK) following maunfacturer’s instructions. The RNA samples were treated with DNase-1 reagent and reverse-transcribed using the SuperScript^®^ VILO^TM^ kit. Real-time quantitative PCR assays were performed using the 7500 Real-Time PCR system (Applied Biosystems, Paisley, UK) for analysing the expression of, *Runx-2* (F: 5′ CCACCACTCACTACCACACG 3′; R: 5′ CAC TCTGGCTTTGGGAAGAG 3′), *Osterix* (F: 5′ AGTTCACCTGCCTGCTCTGT 3′; R: 5′ CTC AAGTGGTCGCTTCTGGT 3′), *Alpl* (F: 5′ CTGACTGACCCTTCGCTCTC 3′; R: 5′ CCA GCAAGAAGAAGCCTTTG 3′), *Osteocalcin* (F: 5′ CTGACCTCACAGATGCCAAG 3′; R: 5′ ACCTTATTGCCCTCCTGCTT 3′), *ERα* (F: 5′ CCTTCTAGACCCTTCAGTGAAGCC 3′; R: 5′ CGAGACCAATCATCAGAATCTCC 3′), *ERβ* (F: 5′ CAGTAACAAGGGCATGGAAC 3′; R: 5′ GTACATGTCCCACTTCTGAC 3′) and *Bmpr2* (F: 5′ GAAACGATAATCATTGCTTTGGC 3′; R: 5′ CCCTGTTTCCGGTCTCCTGT 3′). The expression levels of the genes of interest were normalised to the expression level of the housekeeping gene/endogenous control, *β-actin* (F: 5′ TTGCTGACAGGATGCAGAAG 3′; R: 5′ GTACTTGCGCTCAGGAGGAG 3′). Since the SYBR Green mastermix was used, primer sequences for the genes were validated by dissociation curve/melt curve analysis to rule out the formation of secondary structures, such as primer dimers and hairpin loops. The relative transcript levels of genes of interest were analysed using the comparative C_T_ method (ΔΔC_T_ method). For valid ΔΔC_T_ calculation, the efficiencies of the primers used for amplification of the target sequences in the genes of interest were confirmed to be approximately equal to the efficiency of the primers used for amplification of the target sequence in the housekeeping gene. Fold changes (2^−ΔΔC^_T_) in relative transcript levels were normalised to the endogenous reference (β-Actin) and plotted as bar graphs. The range of gene expression was indicated by 2^−(ΔΔC^_T_^+SD)^ and 2^−(ΔΔC^_T_^−SD)^, where SD: standard deviation of the ΔΔC_T_/ΔC_T_ value. Statistical analyses were performed at the level of ΔC_T_ in order to exclude potential bias due to averaging of data transformed through the 2^−ΔΔC^_T_ equation.

### PCR array

The mouse osteogenesis PCR array (RT^2^Profiler PCR Array–Qiagen, Manchester, UK) was used according to the manufacturer’s protocol to analyse the expression of 84 genes related to skeletal development. The PCR arrays were performed in triplicate using the 7500 Real-Time PCR system and results were analysed using RT^2^Profiler PCR array data analysis version 3.5. Five housekeeping genes, namely *β-Actin, β-2-Microglobulin, Glyceraldehyde-3-phosphate dehydrogenase, β-Glucoronidase, α-Heat shock protein 90*, were included in the array for normalisation of data. The threshold for the fold–change in gene expression was set at 2 and the significance of the difference in gene expression was calculated with the P-value set at 0.05.

### Immunoblotting

Cell lysates were prepared using the radioimmunoprecipitation assay (RIPA) buffer and protein concentrations in the lysates were determined using the Pierce™ BCA protein assay kit (ThermoFisher Scientific, Loughborough, UK). The samples (12 μg protein per well in 15 μl loading volume) were resolved in 10% SDS bisacrylamide gels and electroblotted onto nitrocellulose membranes. The membranes were probed with rabbit anti-BAG-1/C-16 (Santa Cruz Biotechnology, Heidelberg, Germany) and rabbit anti-β-actin primary antibodies, used at 1:200 and 1:3000 dilutions, respectively, followed by the goat anti-rabbit IgG–Peroxidase conjugate secondary antibody (1:5000 dilution). Immobilon Western Chemiluminescent HRP Substrate (Merck Millipore UK Ltd, Watford, UK) was used for signal detection. For digital acquisition of the chemiluminescent signals, blots were imaged using the VersaDoc Imaging System (Bio-Rad Laboratories Ltd., Hemel Hempstead, UK) and data were analysed using Quantity One® 1-D analysis software. Expression of the BAG-1L and BAG-1S proteins were normalised to β-actin.

### Statistical analyses

Statistical analyses were performed using the GraphPad Prism software (GraphPad Software Inc., San Diego, USA). The data is representative of a minimum of three independent experimental repeats and the results are presented as mean ± SD. Depending upon the experimental design, differences between the groups were analysed using paired t-test, unpaired t-test (two-tailed P value) and one-way analysis of variance (ANOVA) with Tukey’s post-hoc test. Results were deemed significant if the probability of occurrence by random chance alone was less than 5% (i.e. P < 0.05).

## Additional Information

**How to cite this article**: Greenhough, J. *et al*. Regulation of osteoblast development by Bcl-2-associated athanogene-1 (BAG-1). *Sci. Rep.* **6**, 33504; doi: 10.1038/srep33504 (2016).

## Supplementary Material

Supplementary Information
