# Supplementary material for: Regulation of osteoblast development by Bcl-2-associated athanogene-1 (BAG-1)
Source: Sci Rep. 2016 Sep 16;6:33504. doi: 10.1038/srep33504 (PMC5025845; doi:10.1038/srep33504)
Supplement: Supplementary Information [file srep33504-s1.pdf]

Regulation of osteoblast development by Bcl-2-associated athanogene-1 (BAG-1)

Joanna Greenhough[1], Emmanouil S. Papadakis[2], Ramsey I. Cutress[2], Paul A. Townsend[3], Richard O.C. Oreffo[1], Rahul S. Tare[1]

[1]Centre for Human Development, Stem Cells and Regeneration, Institute of Developmental Sciences, University of Southampton, Southampton SO16 6YD, United Kingdom

[2]Cancer Research UK Centre Cancer Sciences Unit, Somers Building, University of Southampton, Southampton SO16 6YD, United Kingdom

[3]Institute of Cancer Sciences, Manchester Cancer Research Centre, University of Manchester, Manchester M13 9WL, United Kingdom

<u>Supplementary Information</u>

Supplementary Figure 1

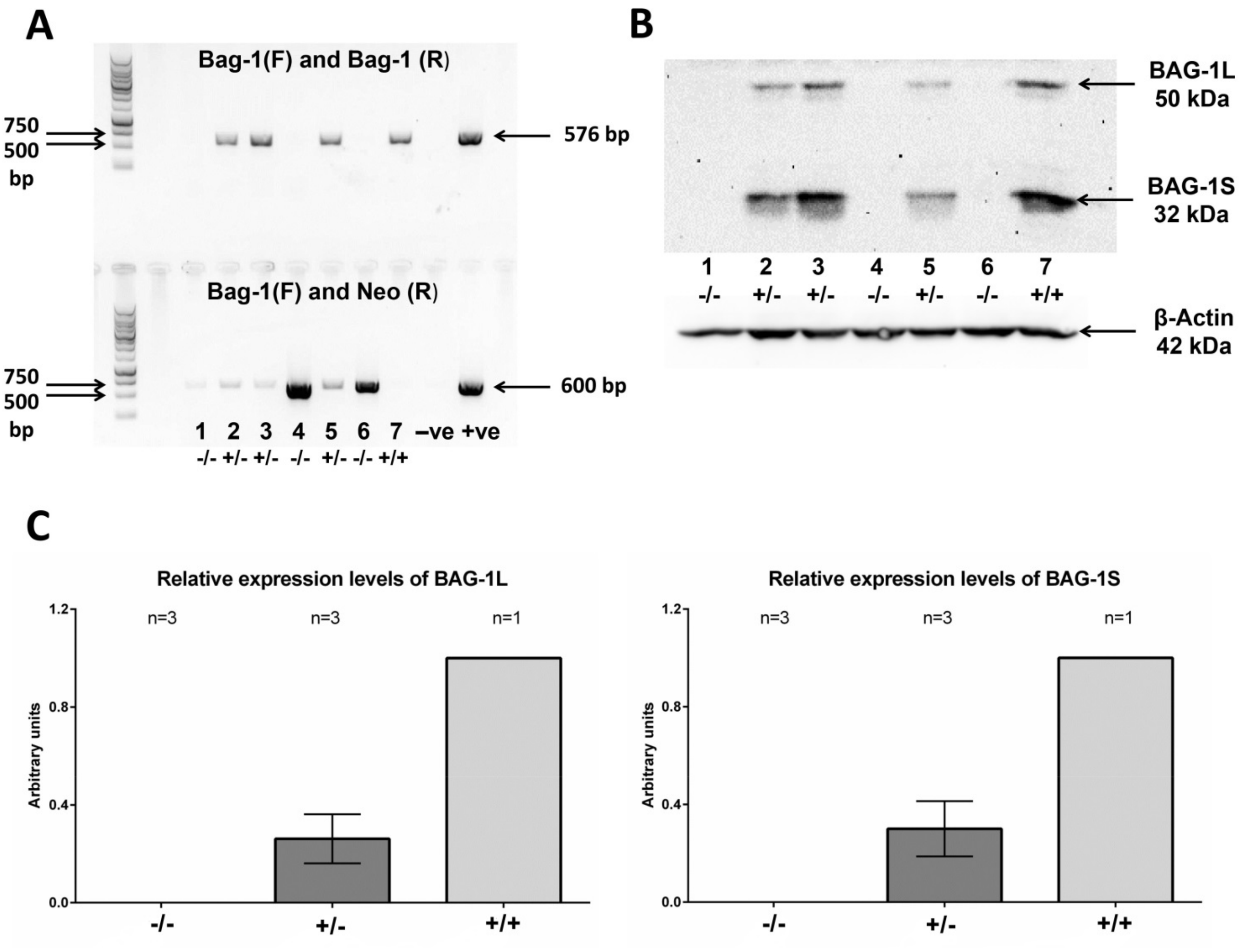

Supplementary Figure 1. Identification of wild-type, *Bag-1* heterozygous and *Bag-1* null littermates by genotyping and immunoblotting. Genomic DNA samples isolated from the tail pieces of the fetuses

were utilized for genotyping, which was performed using a PCR strategy. (A) The results of genotyping E10.5 fetuses (n = 7) are shown as a representative example. The first row of bands shows products (576 bp) of the PCR performed using *Bag-1* (F) and *Bag-1* (R) primers, while the second row of bands shows products (600 bp) of the PCR performed using *Bag-1* (F) and *Neo* (R) primers. The wild-type *Bag-1* allele was detected using the *Bag-1* (F) primer, located upstream of exon 1, and the *Bag-1* (R) primer, located within exon 1 of the *Bag-1* gene. PCR using this primer pair identified *Bag-1*$^{-/-}$ mice (no. 1, 4, 6 - characterised by absence of bands) from *Bag-1*$^{+/+}$ and *Bag*$^{+/-}$ littermates (bands corresponding to 576 bp PCR products observed for both genotypes). The null allele was detected using the *Bag-1* (F) primer and *Neo* (R) primer, located in the Neomycin-resistance gene. PCR using this primer pair distinguished the *Bag-1*$^{+/+}$ mouse (no. 7 - characterised by the absence of a band) from the *Bag-1*$^{+/-}$ and *Bag-1*$^{-/-}$ littermates (no. 2, 3, 5 and no. 1, 4, 6, respectively, characterised by presence of bands corresponding to 600 bp PCR products). Limb buds of E10.5 foetuses were collected to prepare cell lysates for immunoblotting and the immunoblots were probed with the anti-BAG-1 antibody (C16) to confirm the results of genotyping. (B) Bands specific for BAG-1L (50 kDa) and BAG-1S (32 kDa) were observed in lanes for mice 2, 3, 5, 7 and not present in lanes for mice 1, 4, 6, confirming the absence of expression of BAG-1 isoforms in the *Bag-1*$^{-/-}$ mice. Densitometric quantification of the bands was performed to measure the expression levels of the BAG-1L and BAG-1S proteins, data was normalised to β-Actin and plotted in the form of bar graphs. (C) Lower average levels of BAG-1L and BAG-1S proteins were observed in *Bag-1*$^{+/-}$ mice (no. 2, 3, 5), relative to the levels of BAG-1L and BAG-1S proteins (that were assigned an arbitrary value of 1) in the *Bag-1*$^{+/+}$ littermate (no. 7).

Supplementary Figure 2

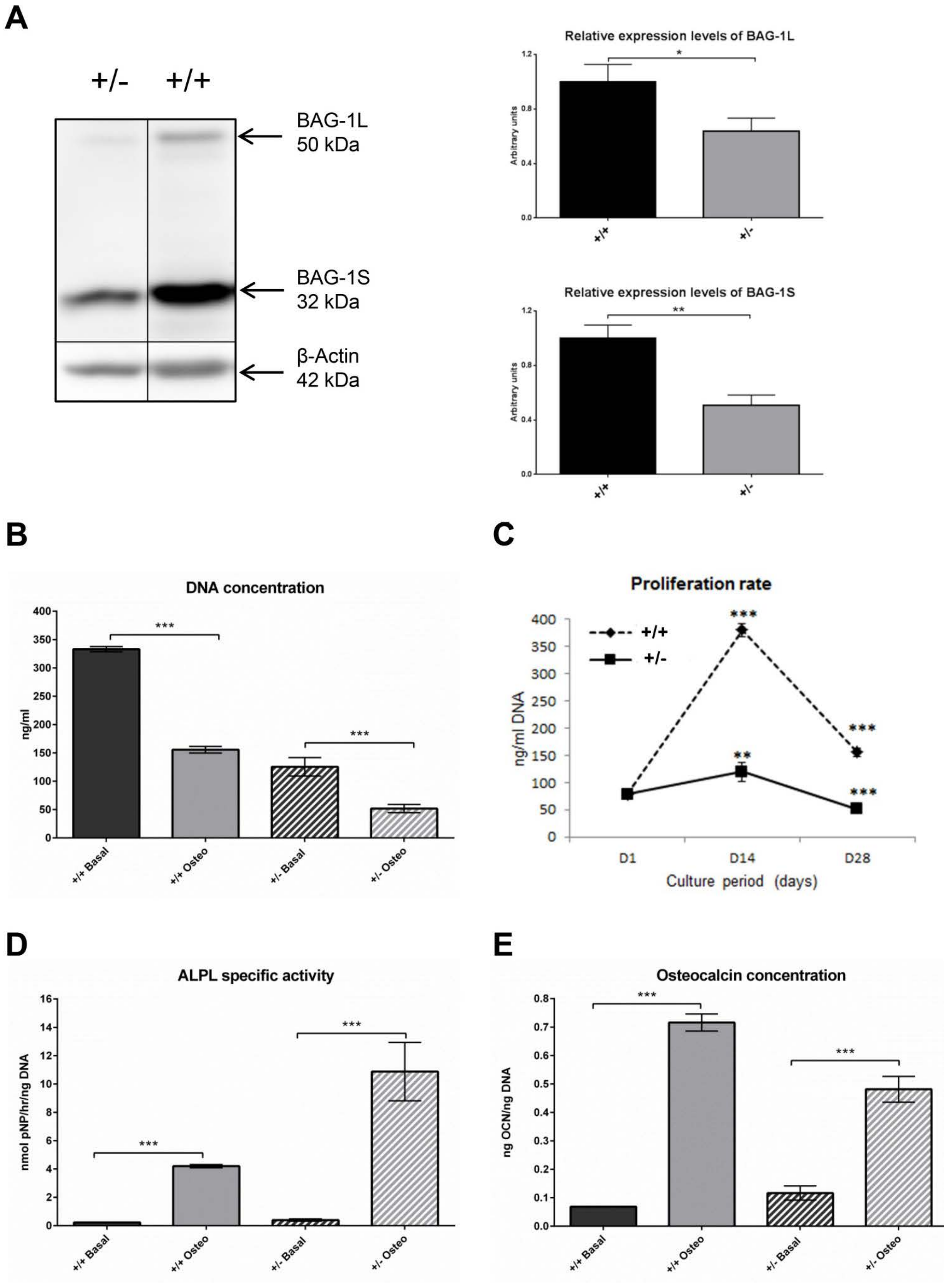

Supplementary Figure 2. Proliferation and BMP-2-stimulated osteogenic differentiation of BMSCs of 14-week-old *Bag-1*$^{+/+}$ and *Bag-1*$^{+/-}$ male mice. (A) Representative immunoblots demonstrating bands for BAG-1L, BAG-1S and β-Actin in day-28 cultures of BMSCs of *Bag-1*$^{+/+}$ and *Bag-1*$^{+/-}$ male mice in basal medium. Densitometric quantification of the bands was performed to measure the expression of the BAG-1L and BAG-1S proteins, data was normalised to β-Actin and plotted in the form of bar graphs. (B) DNA concentrations of day-28 cultures of BMSCs of *Bag-1*$^{+/+}$ and *Bag-1*$^{+/-}$ male mice in basal and osteogenic media. (C) Cell proliferation profiles over the course of 28-day osteogenic cultures of BMSCs of *Bag-1*$^{+/+}$ and *Bag-1*$^{+/-}$ male mice. For statistical analyses, DNA concentrations were compared between days 1 and 14 of culture, and days 14 and 28 of culture. (D) ALPL specific activity and (E) osteocalcin concentration were measured in day-28 cultures of BMSCs of *Bag-1*$^{+/+}$ and *Bag-1*$^{+/-}$ male mice in basal and osteogenic media. Results presented as mean ± SD; n = 3 cultures per group; ***$P<0.001$, **$P<0.01$, *$P<0.05$.

Supplementary Figure 3

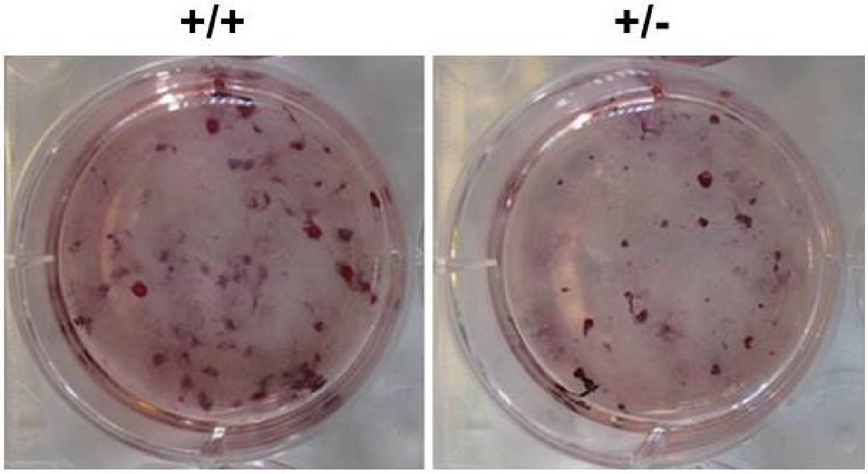

Supplementary Figure 3. Alizarin red-stained colonies in osteogenic cultures of BMSCs of 14-week-old *Bag-1*$^{+/+}$ and *Bag-1*$^{+/-}$ female mice. The number of colonies stained with Alizarin red in the day-28 osteogenic culture of BMSCs of *Bag-1*$^{+/+}$ female mice was higher compared to the day-28 osteogenic culture of BMSCs of *Bag-1*$^{+/-}$ female mice.

Supplementary Figure 4

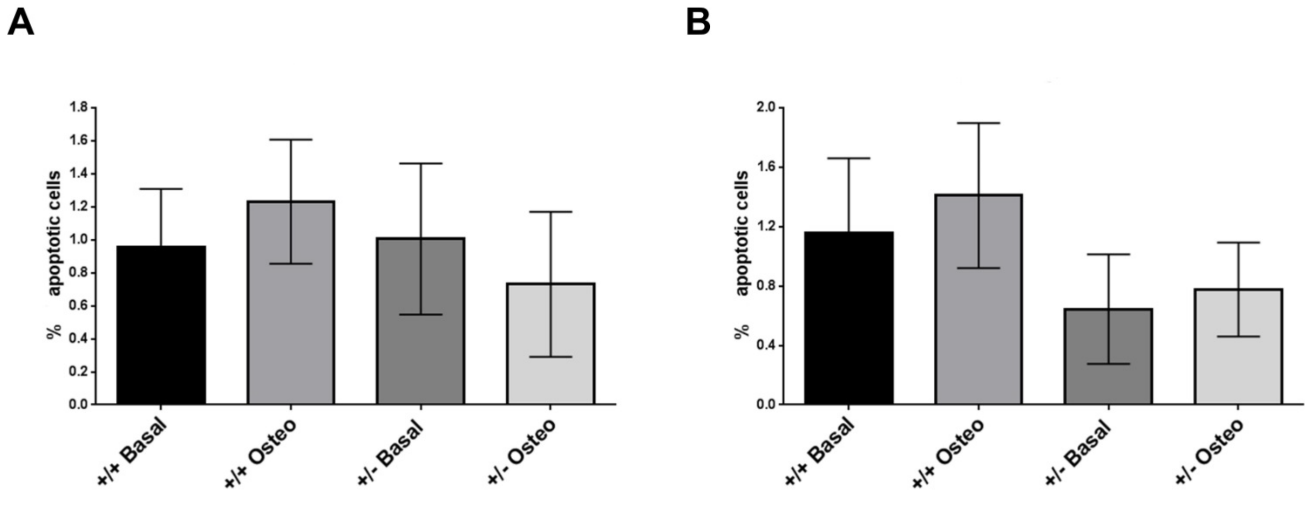

Supplementary Figure 4. Quantification of apoptosis in day-28 BMSC cultures of 14-week-old *Bag-1*$^{+/+}$ and *Bag-1*$^{+/-}$ female and male mice. No differences were observed in the percentages of TUNEL-positive apoptotic cells between day-28 basal and osteogenic cultures of BMSCs of female (A) and male (B) *Bag-1*$^{+/+}$ and *Bag-1*$^{+/-}$ mice. Results presented as mean $\pm$ SD; n = 3 cultures per group.

Supplementary Figure 5

A

Genes upregulated in $F^{+/-}$ vs $F^{+/+}$		
Gene symbol	Fold change (FC)	p-value
Ahsg	11.7483	0.005014
Col14a1	4.4248	0.000427
Col4a1	2.6928	0.000057
Fgf2	3.7754	0.000282
Fn1	2.0112	0.000306
Gdf10	2.1227	0.000829
Mmp8	3.6264	0.000622
Tnfsf11	3.8004	0.000042
Vcam1	2.4633	0.015619

Genes downregulated in $F^{+/-}$ vs $F^{+/+}$		
Gene symbol	Fold change (FC)	p-value
Alpl	-2.0739	0.003856
Bglap	-14.2169	0.000236
Bmp2	-2.9503	0.000007
Bmp3	-658.5576	0.000016
Bmp4	-3.9187	0.000009
Bmp7	-3.6754	0.000372
Col1a1	-6.9066	0.000088
Col1a2	-5.1735	0.0001
Col5a1	-2.4909	0.000269
Comp	-3.1683	0.000034
Ctsk	-2.4984	0.000031
Fgf1	-4.0543	0.000088
Mmp10	-2.2709	0.000847
Mmp2	-2.0047	0.000014
Pdgfa	-2.5567	0.000011
Phex	-511.9712	0.000002
Serpinh1	-3.5068	0.000003
Sost	-7.3678	0.000031
Sp7	-2.2845	0.000037
Spp1	-2.0299	0.000112
Vegfa	-2.6407	0.000091

B

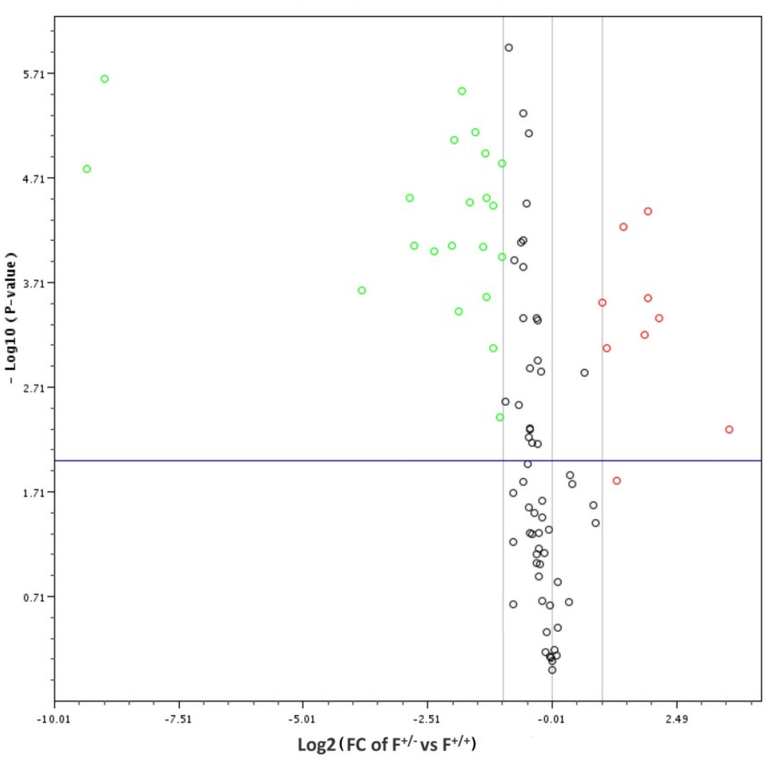

Supplementary Figure 5. Differential expression of genes from the mouse osteogenesis RT² Profiler™ PCR array between BMSC cultures of *Bag-1*$^{+/+}$ and *Bag-1*$^{+/-}$ mice in osteogenic conditions. (A) List of 30 genes that were differentially expressed between day-28 osteogenic cultures of BMSCs of 14-week-old *Bag-1*$^{+/+}$ and *Bag-1*$^{+/-}$ female mice, along with values of fold up/downregulation and statistical significance. (B) Volcano plot, constructed by plotting the negative log of the P-value on the y-axis and log of the fold change between the two groups on the x-axis, enabled rapid visual identification of the 30 differentially expressed genes; red circles represented upregulated genes and green circles represented downregulated genes. The PCR arrays were performed in triplicate, results were analysed using RT^{2}Profiler PCR array data analysis version 3.5, the threshold for the fold–

change in gene expression was set at 2 and the significance of the difference in gene expression was calculated with the P-value set at 0.05.

Supplementary Figure 6

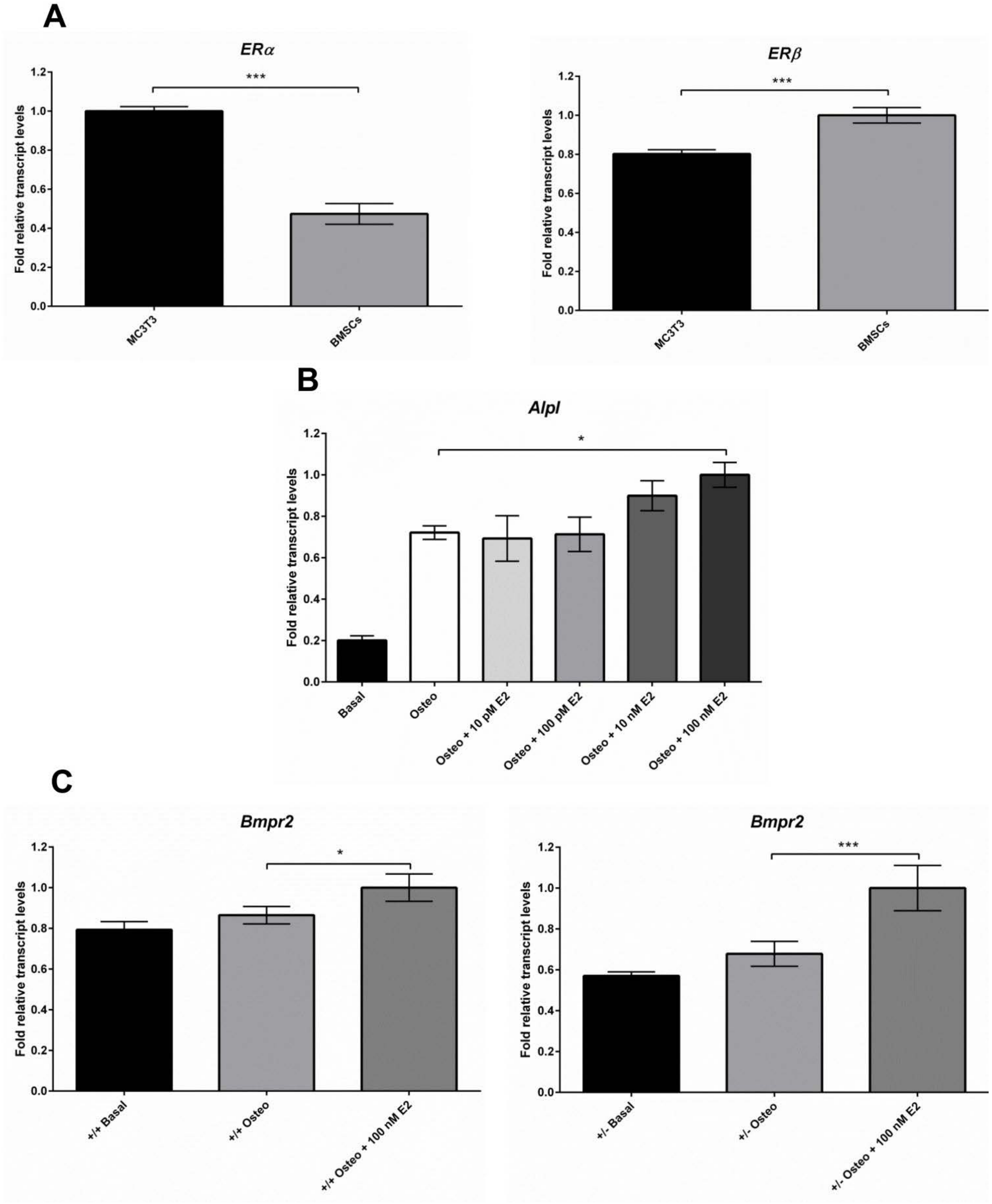

Supplementary Figure 6. Analyses of expression of ERs in day-28 cultures of mouse BMSCs and E2-facilitated BMP-2-directed osteogenic differentiation of BMSCs. (A) Expression of estrogen receptors, namely *ERα* and *ERβ*, was determined by Real-time qPCR in day-28 cultures of BMSCs of wild-type mice in basal medium and compared to murine osteoblast-like MC3T3-E1 cells. Analyses of *Alpl* (B) and *Bmpr2* (C) expression in day-28 cultures of BMSCs in basal, osteogenic and osteogenic media supplemented with different concentrations of E2. Fold changes ($2^{-\Delta\Delta C_T}$) in relative mRNA transcript levels normalised to the endogenous reference (β-Actin) were plotted as bar graphs. The range of gene expression was indicated by $2^{-(\Delta\Delta C_T + SD)}$ and $2^{-(\Delta\Delta C_T - SD)}$, where SD: standard deviation of the $\Delta\Delta C_T/\Delta C_T$ value; n = 3 cultures per group; ***P<0.001, *P<0.05.
